# Leucyl-tRNA synthetase promotes malignant progression in diffuse large B-cell lymphoma by regulating glycolysis via the LRPPRC/HIF-1α/HK2 axis

**DOI:** 10.1007/s13577-025-01267-y

**Published:** 2025-08-07

**Authors:** Weiming Zhang, Nasha Yu, Xiangxiang Song, Xing Zhong

**Affiliations:** 1 Departments of Lymphatic and Hematological Oncology, Jiangxi Cancer Hospital (The Second Affiliated Hospital of Nanchang Medical College), No. 519, Beijing East Road, Nanchang, 330029 Jiangxi China; 2 JXHC Key Laboratory of Tumor Microenvironment and Immunoregulation (Jiangxi Cancer Hospital), Nanchang, 330029 Jiangxi China

**Keywords:** LARS, DLBCL, Glycolysis, LRPPRC, HIF-1α

## Abstract

Diffuse large B-cell lymphoma (DLBCL) is a malignant tumor and research on its therapeutic targets has received increasing attention. It has been reported that Leucyl-tRNA synthetase (LARS) contributes to the growth and migration of non-Hodgkin lymphoma (NHL), yet its effect on DLBCL progression remains to be elucidated. MTT and flow cytometry were carried out to determine the cellular phenotypes of DLBCL cells under LARS overexpression. The differentially expressed proteins (DEPs) were screened by mass spectrometry. ELISA, western blot, and xenograft tumor experiments were implemented to confirm the impact of LARS and the LRPPRC/HIF-1α axis on malignant progression and glycolysis. Dual-luciferase reporter assay and chromatin immunoprecipitation (ChIP) were applied for exploring the underlying mechanism by which LARS was regulated. LARS promoted the malignant phenotypes, such as increasing cell proliferation and inhibiting apoptosis, and enhanced abnormal glycolysis both in vitro and in vivo. Based on mass spectrometry and functional recovery experiments, we found that LARS upregulated LRPPRC expression. More importantly, overexpressing LRPPRC facilitated malignant phenotypes and glycolysis through the elevation of HIF-1α expression, which could be reversed by silenced HIF-1α. Mechanistically, LARS promoted the expression of HIF-1α by activating LRPPRC, which in turn enhanced the transcription of HK2, thereby facilitating malignant progression and abnormal glycolysis. LARS facilitated abnormal glycolysis via the LRPPRC/HIF-1α/HK2 axis, thereby promoting the malignant progression of DLBCL.

**Supplementary Information:**

The online version contains supplementary material available at 10.1007/s13577-025-01267-y.

## Introduction

Diffuse large B-cell lymphoma (DLBCL) is a kind of aggressive non-Hodgkin lymphoma (NHL), accounting for 30%–40% of all cases of NHL [1]. In the past decade, although immunochemotherapy with anthracyclines in combination with R-CHOP had a relatively promising clinical outcome, the overall prognosis of DLBCL as still poor [2]. It has been reported that about 30% of DLBCL patients suffer from intensive side effects, even relapse, due to the heterogeneity and the drug resistance of DLBCL [3, 4]. Thus, there is an urgent need to explore novel therapeutic targets for DLBCL and investigate the underlying mechanism of malignant DLBCL progression.

For energy and development requirements, cancer cells reprogram energy metabolic pathways, for instance, glucose metabolism [5]. It has been proved that glycolysis, the central pathway of glucose metabolism, is the primary metabolic pathway utilized by cancer cells [6]. Cancer cells consume glucose and generate lactic acid (LA) through glycolysis to satisfy their uncontrolled biosynthesis demand. As the substrate of protein synthesis, amino acids also serve as metabolites for cancer development and participate in the regulation of glycolysis [7]. For example, previous studies have shown that high levels of leucine inhibit glycolysis in Walker-256 cancer cells and induce cancer metabolism through oxidative phosphorylation [8]. Leucyl-tRNA synthetase (LARS) is a key enzyme that connects leucine to its isogenous tRNAs for protein translation [9]. According to reports, LARS senses and utilizes leucine for energy generation dependent on the stimulation of glucose, making the trend of metabolism toward the direction of synthesis [10]. Furthermore, it has been proved that LARS overexpression promotes cell migration and growth of lung cancer cells [11]. In breast cancer cells, LARS facilitated cell proliferation yet inhibited apoptosis, thus advancing cancer progression [12]. In short, LARS has been considered a potential therapeutic target in cancer, with a particular focus on glycolysis. However, further studies are required to fully understand the specific mechanism of action of LARS proteins in cancer and its potential clinical value.

## Materials and methods

### Regents

Dulbecco's modified Eagle medium (DMEM, 02-5062EJ), fetal bovine serum (FBS, 16140-071), penicillin–streptomycin (Pen–Strep, 15140-122), and Opti-MEM serum-reduced medium (31985-070) were purchased from Gibco (Semitic, NY, USA). Lipofectamine^TM^ 2000 (Lipo 2000, 11668-019) was obtained from Invitrogen (Waltham, MA, USA). Puromycin was obtained from Solarbio (Beijing, China). Enhanced chemiluminescence (ECL, BL520B) kit was purchased from Biosharp (Hefei, China). MTT reagent (M2128) and DMSO (D2650) were obtained from Sigma-Aldrich (St. Louis, MO, USA); SYBR (Q311‐02/03) was from Vazyme (Nanjing, China); citrate buffer (G1202), DAB solution (G1212), and hematoxylin (G1004) were from Servicebio (Wuhan, China).

### Cells

The two human DLBCL cell lines, SU-DHL-2 (YDT-0623) and U2932 (YDT-0681), were purchased from INDIT (Hangzhou, China). Cells were seeded in a 25 cm^2^ cell culture flask and cultured in DMEM medium containing 10% FBS and 1% Pen–Strep with 5% CO_2_ at 37°C.

### Transient transfection

All siRNA constructs (siR-LARS, siR-LRPPRC, siR-HIF-1α, and siR-NC) were obtained from Hippo Bio (Huzhou, China), while expression plasmids (pcDNA3.1-LARS, pcDNA3.1-LRPPRC, pcDNA3.1-HIF-1α, and empty pcDNA3.1 vector) were purchased from Youbio (Hunan, China). For transient transfections, SU-DHL-2 and U2932 cells were seeded in six-well plates at 70–80% confluence prior to transfection. siRNA transfections were performed using 100 pmol of siRNA (final concentration 50 nM) complexed with 5 μL of Lipofectamine 2000 in Opti-MEM serum-reduced medium, representing a Lipofectamine:siRNA ratio of 1:1 (v/v), with cells harvested 24 h post-transfection for analysis. Plasmid transfections utilized 4 μg of DNA combined with 10 μL of Lipofectamine 2000 (ratio 2.5:1 (v/w)), followed by 24 h of incubation before assessment. Transfection efficiency was validated by western blot analysis.

### Construction of LARS stably overexpressed SU-DHL-2 cells

For the construction of LARS stably overexpressed SU-DHL-2 cells, the LARS-overexpressed lentivirus (Lenti-LARS) and the corresponding control lentivirus (Lenti-control) were purchased from GeneChem (Shanghai, China). When the confluence of SU-DHL-2 cells reached 70%, cells were co-incubated with the Lenti-LARS or Lenti-control (at a titer of 1.0 ×108 Tu·ml-1) for 48 h. Then 1 μg/mL puromycin was utilized for screening.

### Animal models

Twelve male BALB/c nude mice (4–5 weeks old) purchased from SLAC Laboratory Animal Co., Ltd (Shanghai, China) were housed under specific pathogen-free (SPF) conditions with controlled temperature (22 ± 1 °C), humidity (50 ± 10%), and a 12-h light/dark cycle. Mice were randomly divided into two groups (*n* = 6 per group) and subcutaneously injected with SU-DHL-2 cells stably overexpressing LARS or control vector (5×10^6^ cells suspended in 100 μL PBS). Tumor growth was monitored every 3 days by caliper measurements (volume calculated as *V = 0.5 × length × width^2^*). When tumors reached the humane end point (≥2000 mm^3^) or showed signs of distress (e.g., >20% weight loss, ulceration), mice were euthanized by cervical dislocation. Excised tumors were measured for volume and weight. The animal experiments were conducted in accordance with the protocols approved by the Medical Ethics Committee of Jiangxi Cancer Hospital (Approval No. 2025ky007). All procedures strictly followed the institutional guidelines for animal welfare and the ARRIVE guidelines.

### Western blot (WB)

The western blot procedures were performed as previously described with the following modifications [13]. Total protein was extracted from tumor tissues and SU-DHL-2 and U2932 cells using RIPA lysis buffer supplemented with protease and phosphatase inhibitors. Protein samples (20–30 μg per lane) were separated by 10% SDS-polyacrylamide gel electrophoresis and transferred to PVDF membranes (0.45 μm pore size). Membranes were blocked with 5% bovine serum albumin (BSA) in TBST (Tris-buffered saline with 0.1% Tween-20) for 1 h at room temperature to prevent nonspecific binding.

Primary antibodies were diluted in 5% BSA/TBST according to the concentrations listed in Table 1 and incubated with membranes overnight at 4°C. After washing, membranes were probed with species-matched horseradish peroxidase (HRP)-conjugated secondary antibodies (anti-rabbit IgG, CST 7074P2, 1:2000; anti-mouse IgG, CST 7076P2, 1:2000) in 5% BSA/TBST for 1 h at room temperature. Protein bands were visualized using an enhanced chemiluminescence (ECL) kit with exposure times ranging from 1 to 90 s (optimized for each target protein) on a ChemiDoc imaging system. Image analysis was performed using Image Lab software (Bio-Rad).

**Table 1:** Details of antibodies

Name	Brand	Number	Dilution rate	Applications
β-Actin	CST	4970	1:5000	WB
LARS	Proteintech	21146-1-AP	1:1000	WB
HK2	CST	2867T	1:1000	WB, IHC
GLUT1	Proteintech	81463-1-RR	1:5000	WB, IHC
LRPPRC	Proteintech	67679-1-lg	1:5000	WB
HIF-1α	abcam	ab179483	1:1000	WB

### MTT assay

The MTT assay was carried out to detect the cell proliferation of SU-DHL-2 and U2932 after transfection treatment. Cells were seeded into a 96-well plate at a density of 2×10^3^ cells/well and cultured for 0, 24, 48, and 72 h. The MTT reagent was diluted to 1 mg/mL with phosphate buffer solution (PBS) and then added to the 96-well plate containing cell suspensions (50 μL per well) for co-incubation for 3 h at 37°C. After the addition of DMSO (150 μL per well) and the dissolution of crystallization, the results were quantified utilizing a microplate reader (Molecular Device, California, USA) at the absorbance of 570 nm.

### Flow cytometry

Flow cytometry was applied to determine the apoptosis of SU-DHL-2 and U2932 cells after transfection treatment. The procedures were completed using the Annexin V–FITC/PI Apoptosis Detection Kit (4A Biotech, Beijing, China, FXP018) according to the manufacturer's protocol. Then the results were detected with a flow cytometer.

### LA detection assay

The expression of LA was determined using the human LA enzyme-linked immunosorbent assay (ELISA) detection kit (MEIMIAN, Jiangsu, China, 12907) according to the producer’s instructions. Then the OD value of the samples was examined with the microplate reader at the absorbance of 450 nm.

#### Immunohistochemistry (IHC)

The IHC assay was implemented to detect the expression of HK2 and GLUT1 in the tumor tissues. The tissues cut into sections were de-paraffinized, and then successively soaked in citrate buffer for antigen retrieval and in 3% H_2_O_2_ for endogenous peroxidase removal. Subsequently, sections were blocked with 3% BSA and then incubated with antibodies specific for HK2 and GLUT1 overnight. After three washes with PBS, slides were incubated in secondary antibodies. Then the sections were stained with the DAB solution and counterstained with hematoxylin. The results were observed with a microscope.

#### Bioinformatics

Bulk RNA-seq data (GSE25297) were analyzed using limma in R, with log2(TPM) transformed values compared between DLBCL (*n* = 7) and normal B cells (*n* = 7) via t-test. scRNA-seq data (GSE182434) were processed with Seurat v5.2.1: after quality control, 2000 variable genes were used for PCA (30 PCs) and UMAP clustering (resolution = 0.5). B cells (CD19+/MS4A1+) were stratified into the LARS+ (TPM≥1) and LARS− (TPM<1) groups for proportion (Chi-square test) and LRPPRC expression (Wilcoxon test) analyses. All visualizations used ggplot2/ggpubr in R.

The survival curve was generated based on The Cancer Genome Atlas (TGCA) database utilizing GraphPad Prism 6.0. The differentially expressed genes (DEGs) between the LARS high- and low-expression samples were obtained using DESeq2 R Package 1.22.2 and exhibited using the volcano plot. Based on the information of DEGs, Kyoto Encyclopedia of Genes and Genomes (KEGG) and Gene Ontology (GO) enrichment analyses were carried out by Ensembl BioMart101. The protein of LARS-overexpressed SU-DHL-2 cells was extracted and mass spectrometry was performed by LC-BIO (Hangzhou, China) to obtain the differentially expressed proteins (DEPs). Then the results were presented as the heatmap, which was generated by R version 3.6.3.

#### Dual-luciferase reporter assay

The putative HIF-1α binding site within the HK2 3'UTR region and its mutated variant were amplified and cloned into the PGL3-basic vector (Promega, Madison, WI, USA). Constructs (HK2-3'UTR-WT and HK2-3'UTR-Mut) along with empty PGL3 vector were co-transfected with pRL-TK Renilla luciferase control vector into SU-DHL-2 cells using Lipofectamine 2000. Cells were harvested 24 h post-transfection, and firefly luciferase activity was measured using the Dual-Luciferase Reporter Assay System (Promega, Madison, WI, USA) and normalized to Renilla luciferase activity. All transfections were performed in three independent biological replicates (*n* = 3).

#### Chromatin immunoprecipitation (ChIP)

The chromatin immunoprecipitation (ChIP) assay was performed using the ChIP-Seq High Sensitivity Kit (Abcam, ab185908) with the following optimized conditions. Briefly, 1×10^7^ SU-DHL-2 or U2932 cells per immunoprecipitation reaction were cross-linked with 1% formaldehyde for 10 min at room temperature, followed by quenching with 125 mM glycine. Chromatin was sheared to 200–500 bp fragments using a Bioruptor Pico sonicator (30 s ON/30 s OFF, 15 cycles).

Immunoprecipitation was performed overnight at 4 °C using 5 μg of anti-HIF-1α antibody (Abcam, ab179483) with protein A/G MagPoly beads. Normal rabbit IgG (Abcam, ab172730) served as negative control. After reverse cross-linking and DNA purification, the enriched DNA fragments were analyzed by qPCR using the following primers specific to the HK2 promoter region:

forward: 5'-TTCCGTCCCAGCCTTTAGCC-3',

reverse: 5'-TCATCGCTCACGGCTCGC-3'.

The PCR amplification was performed with SYBR Green Master Mix under the following conditions: 95 °C for 10 min, followed by 40 cycles of 95 °C for 15 s and 60 °C for 1 min. All ChIP-qPCR results were normalized to input DNA and expressed as fold enrichment relative to the IgG control.

#### Statistical analysis

Every experiment was repeated at least three times, and the typical data were selected and analyzed with GraphPad Prism 6.0. *t*-test was used for comparison between two groups, and one-way ANOVA for that of multiple groups. *p*<0.05 was regarded as statistically significant.

## Results

### LARS is highly expressed in DLBCL and associated with glycolysis

**Fig. 1:**
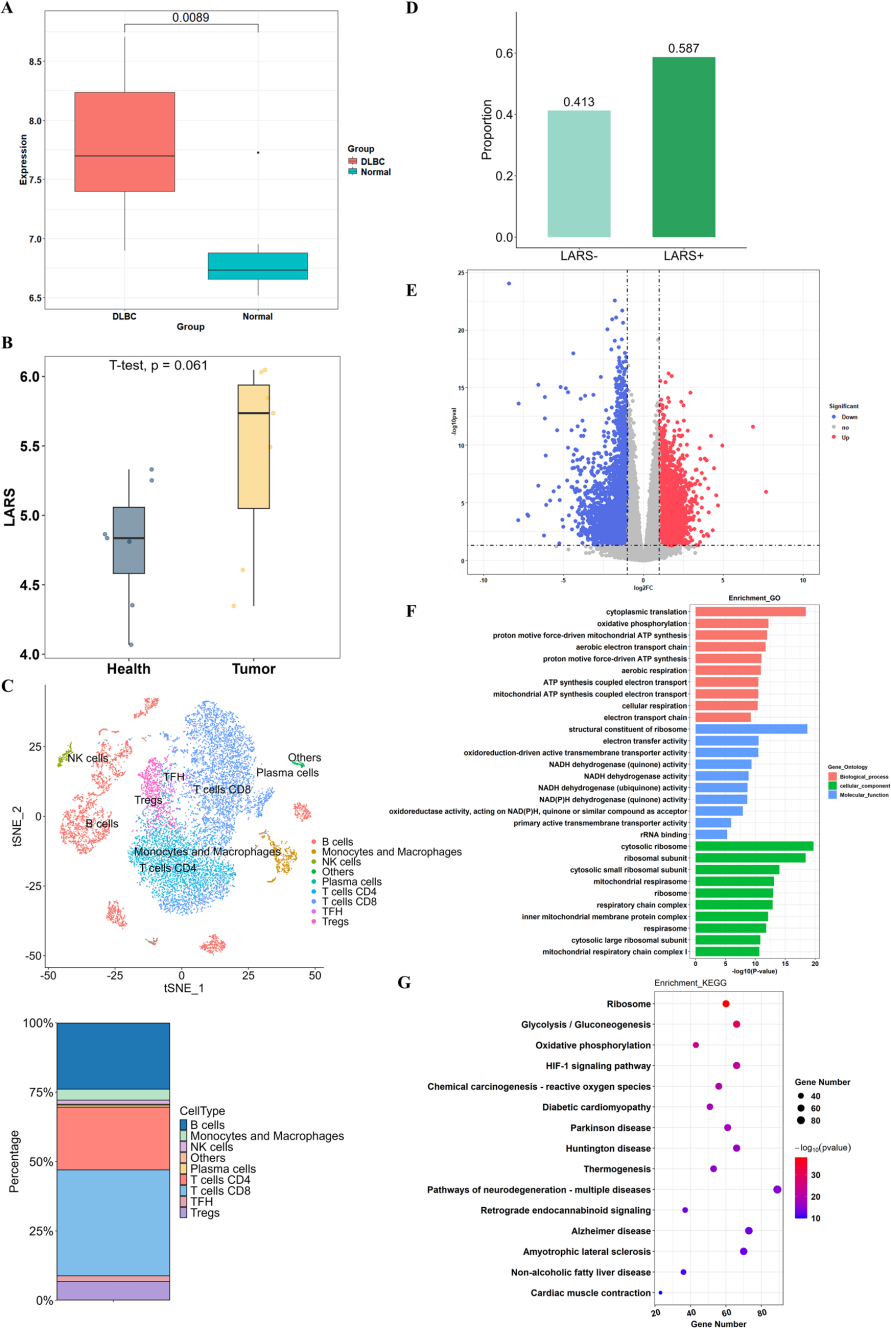
LARS was highly expressed in DLBCL and was associated with glycolysis. (**A**) The expression levels of LARS in DLBCL and normal groups. (**B**) Comparative analysis of LARS mRNA expression levels from GEO dataset GSE25297, showing DLBCL groups (Tumor, *n* = 7) versus adjacent normal samples (Health, *n* = 7). Data are presented as mean ± SEM. The p-value (*p* = 0.061) was calculated by two-tailed t-test of log2 (TPM+1) transformed data. (**C**) UMAP projection of eight major cell populations in DLBCL, color-coded by cell type (upper panel). Percentages indicate relative abundance of each subset (lower panel), Including: B cells (23.91%), CD8^+^ T cells (38.16%), CD4^+^ T cells (22.65%), Tregs (6.77%), monocytes/macrophages (3.95%), TFH cells (2.05%), NK cells (1.48%), and plasma cells (0.71%). Data were derived from integrated analysis of four DLBCL samples (GSE182434) using Seurat v5.2.1. (**D**) B cells from DLBCL samples were classified into LARS+ and LARS− subgroups based on LARS expression. Data derived from scRNA-seq (GSE182434). (**E**) Volcano plots were generated by the R package and showed the distribution of DEGs, the x-axis is log2 of fold change, and the y-axis is the P value (−log10). (**F**&**G**) The biological processes and pathways in which DEGs were enriched were identified by GO and KEGG enrichment analysis

Research has shown that LARS plays a role in tumor development through multiple pathways, and high levels of LARS expression are associated with tumor proliferation, metastasis, and poor prognosis [14]. As shown in Fig. 1A, the expression level of LARS was higher in the DLBCL group compared to normal tissues (*P* = 0.0089). This highlights the significant clinical significance of LARS. Analysis of bulk RNA-seq data from GSE25297 (7 DLBCL tumors vs. 7 adjacent healthy controls) revealed a trend of higher LARS expression in tumor tissues compared to adjacent healthy samples, though this difference did not reach statistical significance (*P* = 0.061, Fig. 1B). To further characterize the tumor microenvironment, we performed single-cell RNA sequencing on four pre-processed DLBCL samples (GSE182434). Following quality control and integration with Seurat (v5.2.1), we specifically analyzed the B-cell compartment, which constituted 23.91% of immune infiltrates (Fig. 1C). Subclustering of B cells revealed a striking predominance of LARS+ B cells (58.7%) over LARS− cells (41.3%, Fig. 1D). To investigate the impact of LARS on DLBCL, differentially expressed genes (DEGs) between the LARS high- and low-expression samples in the database were identified, with 2657 genes upregulated and 3302 genes downregulated (Fig. 1E). The results derived from Gene Ontology (GO) and Kyoto Encyclopedia of Genes and Genomes (KEGG) enrichment analyses demonstrated that these DEGs were mainly involved in glycolysis/gluconeogenesis, oxidative phosphorylation, cellular respiration, chemical carcinogenesis–reactive oxygen species, and hypoxia-inducible factor 1 (HIF-1) signaling pathway (Fig. 1F&G). This suggested LARS-mediated metabolic reprogramming may confer selective advantages in DLBCL pathogenesis.

### LARS promoted malignant progression and abnormal glycolytic metabolism in DLBCL

**Fig. 2:**
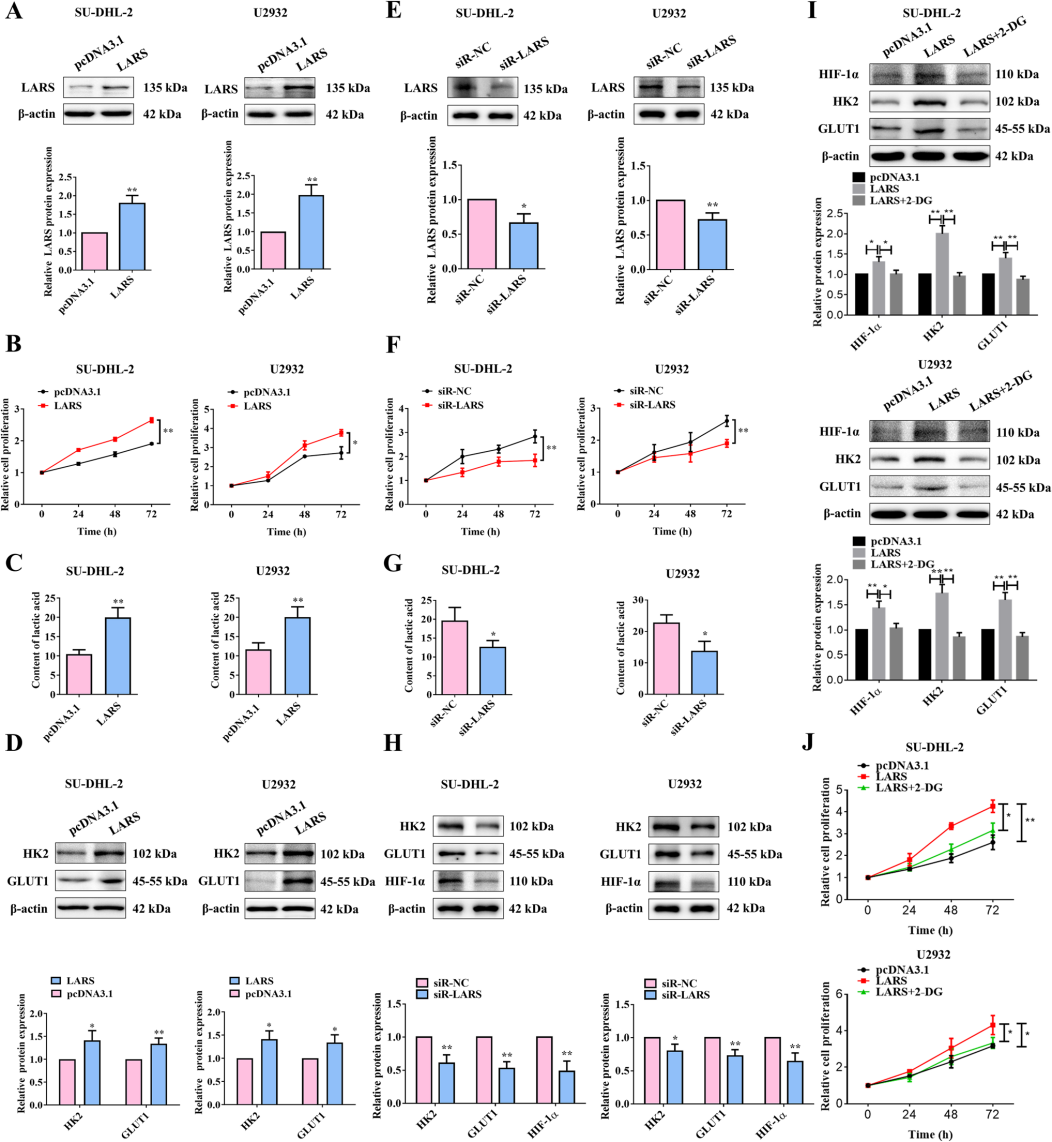
LARS promoted the malignant phenotypes and abnormal glycolytic metabolism in DLBCL cells. (**A**) SU-DHL-2 and U2932 cells were transfected with LARS-overexpressed plasmid or the empty pcDNA3.1 vector. The overexpressing efficiency was verified by WB assays (*n* = 3). (**B**) The cell proliferation of the two DLBCL cell lines overexpressing LARS was detected by the MTT assays (*n* = 3). (**C**) The content of LA was examined by ELISA (*n* = 3). (**D**) The expression of glycolysis-related proteins (HK2 and GLUT1) was determined by WB assays (*n* = 3). β-Actin served as the loading internal control. (**E**) SU-DHL-2 and U2932 cells were transfected with siR-LARS, and the silencing efficiency was verified by the WB assays (*n* = 3). (**F**) The cell proliferation of the two DLBCL cell lines transfected with siR-LARS was detected by the MTT assays (*n* = 3). (**G**) The content of LA was examined by ELISA (*n* = 3). (**H**) The expression of glycolysis-related proteins (HK2, GLUT1, and HIF-1α) was determined by WB assays (*n* = 3). β-Actin served as the loading internal control. (**I**) The expression of HIF-1α, HK2, and GLUT1 in the LARS-overexpressed DLBCL cell lines (SU-DHL-2 and U2932 cells) combined with 2-DG treatment (2.5 mM) was detected by the WB assays. β-Actin served as the loading internal control (*n* = 3). (**J**) The cell proliferation of the two DLBCL cell lines overexpressing LARS combined with 2-DG treatment (2.5 mM) was detected by MTT assays (*n* = 3). Statistical analysis was performed utilizing a T-test for two-group comparison and one-way ANOVA for multiple-group comparison. *p<0.05, **p<0.01

To further explore the mechanism of LARS in DCLBL, we constructed LARS-overexpressed SU-DHL-2 and U2932 cells (Fig. 2A), and subsequently examined the malignant phenotypes and glycolysis-related indexes. The results revealed that overexpression of LARS promoted cell proliferation (Fig. 2B) and inhibited apoptosis of the two DLBCL cells (Fig. S1A). Besides, overexpression of LARS upregulated the content of lactic acid (LA, a product of glycolysis) in cells (Fig. 2C). Hexokinase-2 (HK2) is the first enzyme in the glycolytic pathway and serves as the rate-limiting enzyme in glycolysis [15]. Glucose transporter 1 (GLUT1), as a glucose transporter, facilitates the transport of glucose into the cell, thereby participating in the progression of glycolysis to provide energy and raw materials for biosynthesis [16]. Overexpression of LARS raised the expression levels of HK2 and GLUT1 (Fig. 2D), illustrating the promoting effect of LARS on glycolysis. LARS was knocked down in SU-DHL-2 and U2932 cells using siRNA to investigate the tumor-promoting role of LARS in DLBCL (Fig. 2E). In contrast to the oncogenic effects of LARS overexpression, silencing of LARS led to significant suppression of cell proliferation (Fig. 2F), confirming its essential role in DLBCL cell survival. At the metabolic level, LARS knockdown resulted in decreased LA accumulation (Fig. 2G), accompanied by reduced expression of the glycolytic regulators HK2, GLUT1, and hypoxia-inducible factor 1-alpha (HIF-1α) (Figure 2H). These findings demonstrate that LARS deficiency impairs glycolytic metabolism and attenuates the malignant behavior of DLBCL cells, providing compelling evidence that LARS drives tumor progression through metabolic reprogramming.

To further validate the impact of LARS on glycolysis, 2-deoxy-D-glucose (2-DG), a glycolysis antagonist, was applied to DLBCL cells. Overexpression of LARS significantly elevated the protein levels of key glycolysis regulators, including HK2, GLUT1, and HIF-1α, which were reversed upon 2-DG treatment (Fig. 2I). HIF-1α enhances glycolysis under hypoxia by upregulating glycolytic enzymes and lactate transporters [17]. Additionally, the proliferation-enhancing effect induced by overexpression of LARS was significantly mitigated upon 2-DG treatment (Fig. 2J). This emphasized the pivotal function of LARS in advancing malignant progression and abnormal glycolysis of DLBCL.

**Fig. 3:**
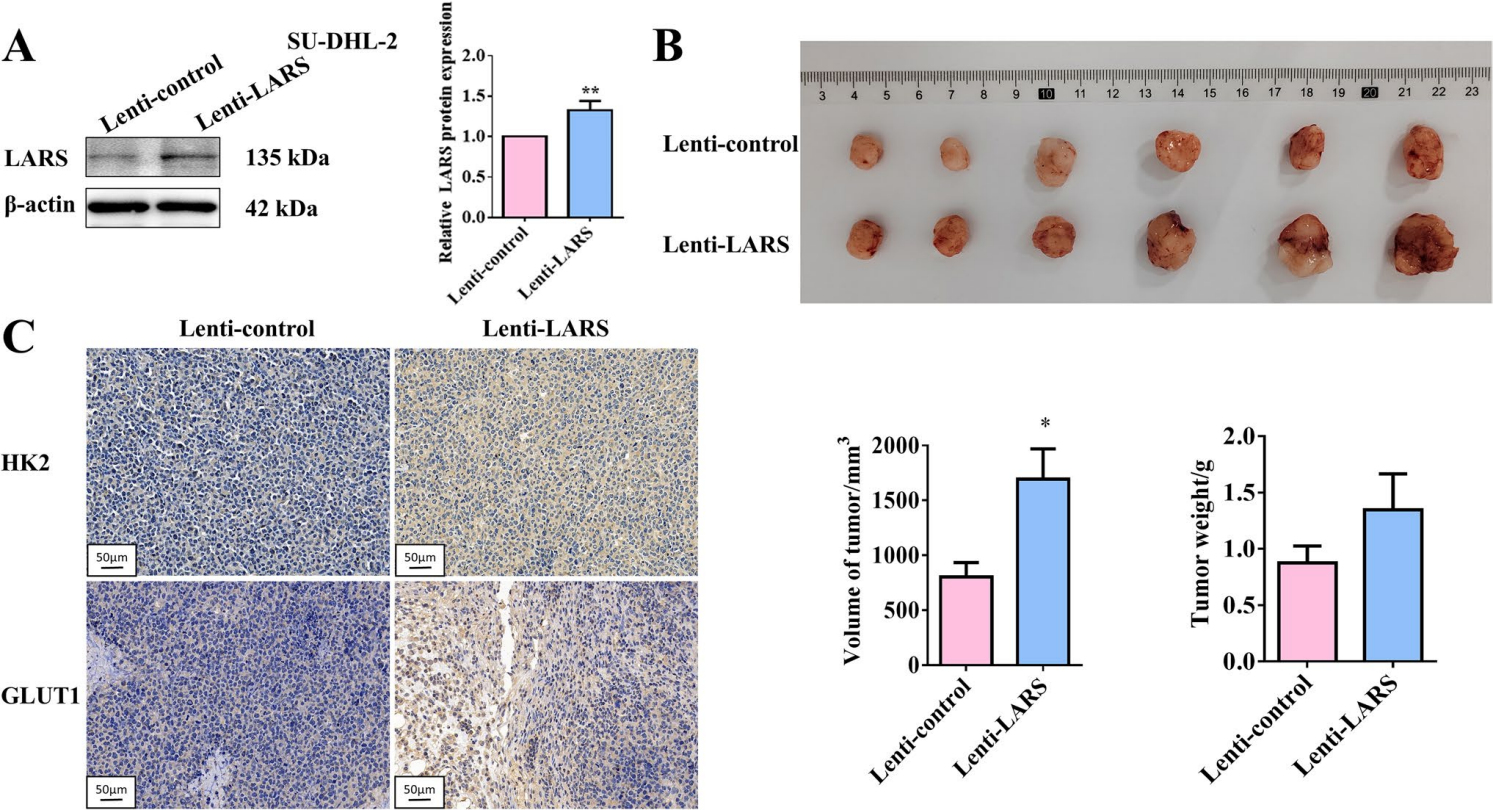
LARS promoted the tumor growth of DLBCL in vivo*.* (**A**) SU-DHL-2 cells were transfected with Lenti-LARS or Lenti-control, and then the overexpressing efficiency was verified by WB assays. β-Actin served as the loading internal control (*n* = 6). (**B**) The mice were subcutaneously injected with LARS stably overexpressed or the control SU-DHL-2 cells. After tumors grew to the appropriate size, tumor tissues were isolated from the mice and then the volume and weight of the tumors were recorded (*n* = 6). (**C**) The expression levels of HK2 and GLUT1 were detected by the IHC assays (*n* = 6). Statistical analysis was performed utilizing a T-test for two-group comparison and one-way ANOVA for multiple-group comparison. *p<0.05, **p<0.01

SU-DHL-2 cells overexpressing LARS were further used in xenograft tumor experiments to assess the impact of LARS on tumor growth in vivo (Fig. 3A). The results demonstrated that overexpression of LARS facilitated tumor growth and simultaneously promoted the expression levels of HK2 and GLUT1 (Fig. 3B&C). To sum up, LARS contributed to malignant progression and abnormal glycolysis metabolism of DLBCL.

### LARS facilitated the malignant phenotypes based on glycolysis by upregulating the LRPPRC expression in DLBCL cells

**Fig. 4:**
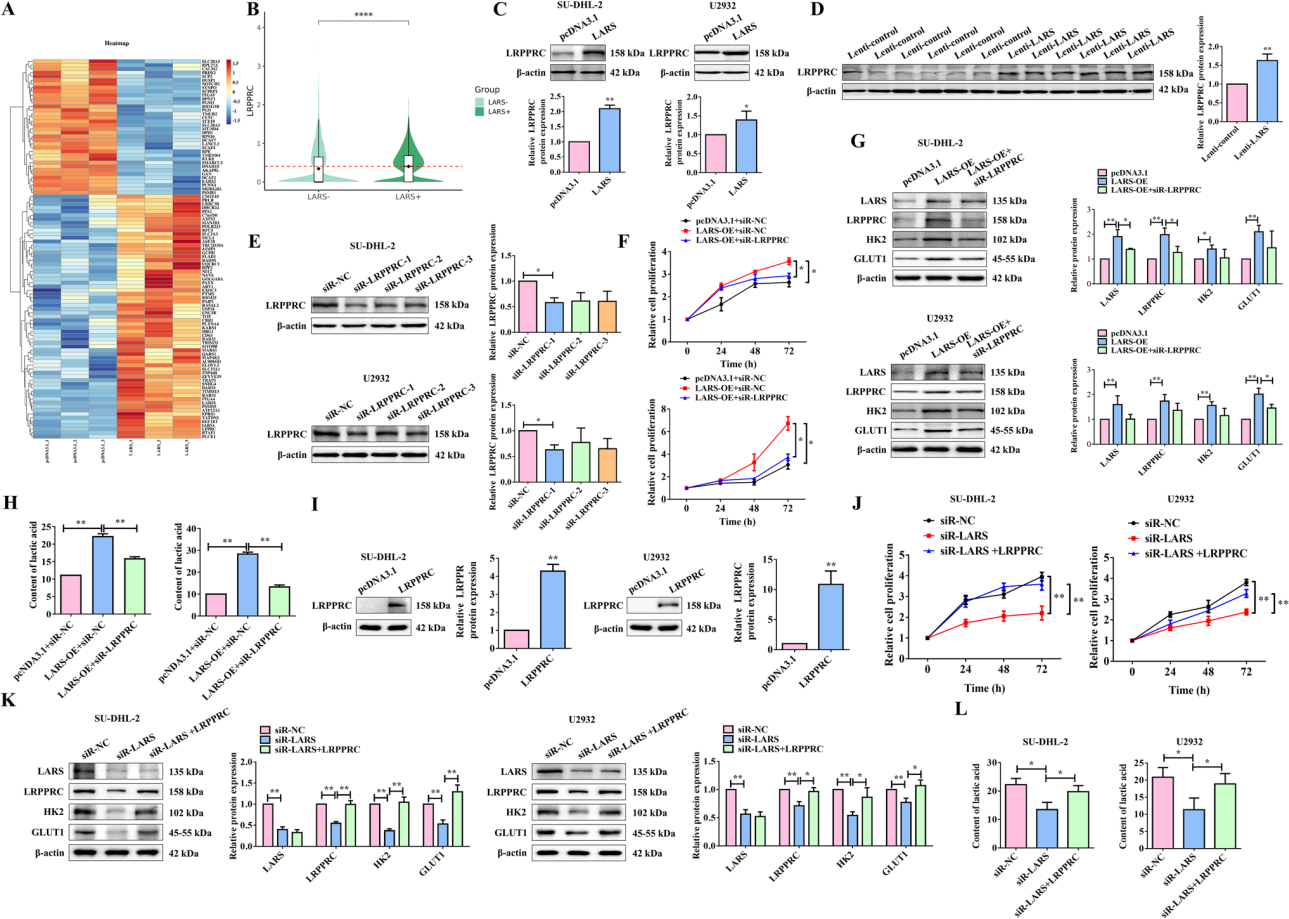
LARS facilitated the malignant phenotypes based on glycolysis by upregulating the LRPPRC expression in DLBCL cells. (**A**) The protein samples of LARS-overexpressing SU-DHL-2 cells were extracted and the DEPs screened by mass spectrometry. The results are presented as the heatmap. (**B**) Violin plot showing LRPPRC expression levels in LARS+ versus LARS− B cells. Central boxplots depict interquartile ranges with median lines. Statistical significance (****p < 0.001) was calculated using Wilcoxon rank-sum test. (**C**) The protein expression level of LRPPRC in SU-DHL-2 and U2932 cells overexpressing LARS was detected by WB assays (*n* = 3). (**D**) The protein expression level of LRPPRC in tumor tissues (from Figure 3B) was examined by the WB assays (*n* = 3). (**E**) SU-DHL-2 and U2932 cells were transfected with three types of siR-LRPRC or siR-NC, and the silencing efficiency was verified by the WB assays (*n* = 3). (**F**) The cell proliferation of the LARS-overexpressed DLBCL cell lines (SU-DHL-2 and U2932 cells) transfected with siR-LRPPRC or siR-NC was examined by MTT assays (*n* = 3). (**G**) The protein expression levels of LARS, LRPPRC, HK2, and GLUT1 were detected by WB assays. β-Actin served as the loading internal control (*n* = 3). (**H**) The content of LA was examined by ELISA (n = 3). (**I**) SU-DHL-2 and U2932 cells were transfected with LRPPRC-overexpressed plasmid or the empty pcDNA3.1 vector. The overexpressing efficiency was verified by WB assays (*n* = 3). (**J**) MTT assay was performed to compare proliferation between LRPPRC-overexpressing and control DLBCL cell lines (SU-DHL-2 and U2932) following transfection with either siR-LARS or siR-NC (*n* = 3). (**K**) The protein expression levels of LARS, LRPPRC, HK2, and GLUT1 were detected by WB assays. β-Actin served as the loading internal control (*n* = 3). (**L**) The content of LA was examined by ELISA (*n* = 3). Statistical analysis was performed utilizing a T-test for two-group comparison and one-way ANOVA for multiple-group comparison. *p<0.05, **p<0.01

Given that the growth and progression of cancers are dependent on glycolysis for energy requirement [18], we wondered how the promoting effect of LARS on DLBCL progression was achieved by regulating glycolysis. To explore further, we obtained the differentially expressed proteins (DEPs) in SU-DHL-2 cells overexpressing LARS by mass spectrometry (Fig. 4A). Among them, leucine-rich pentatricopeptide repeat-containing protein (LRPPRC) caught our attention. Single-cell RNA-seq analysis revealed significantly higher expression of LRPPRC in LARS+ B cells compared to LARS− B cells (Fig. 4B). This differential expression pattern suggests a potential functional link between LARS-mediated metabolic regulation and LRPPRC gene expression in DLBCL B cells. It has been reported that LRPPRC, a multifunctional protein involved in energy metabolism, has a positive regulatory effect on glycolysis [19]. As shown in Fig. 4C, D, overexpression of LARS resulted in an increase in the protein expression of LRPPRC both in vitro and in vivo. Consequently, LRPPRC was silenced in SU-DHL-2 and U2932 cell lines for further cellular phenotype experiments (Fig. 4E). The results demonstrated that the impact of LARS overexpression on promoting cell proliferation and inhibiting apoptosis was reversed by silenced LRPPRC (Fig. 4F&S1B). Likewise, silencing of LRPPRC reversed LARS overexpression-induced elevated expression of HK2 and GLUT1, as well as increased content of LA (Fig. 4G&H). To further confirm the functional relationship between LARS and LRPPRC, we performed rescue experiments in LARS-knockdown cells (Fig. 4I). As expected, overexpression of LRPPRC attenuated the suppression of cell proliferation induced by LARS knockdown (Fig. 4J). Moreover, the decreased levels of HK2, GLUT1, and LA caused by LARS knockdown were significantly reversed after LRPPRC overexpression (Fig. 4K&L). These results indicated that LARS facilitated glycolysis by upregulating the LRPPRC expression, thus promoting the malignant phenotypes of DLBCL cells.

### LARS promotes malignant progression by regulating glycolysis via the LRPPRC/HIF-1α/HK2 axis

**Fig. 5:**
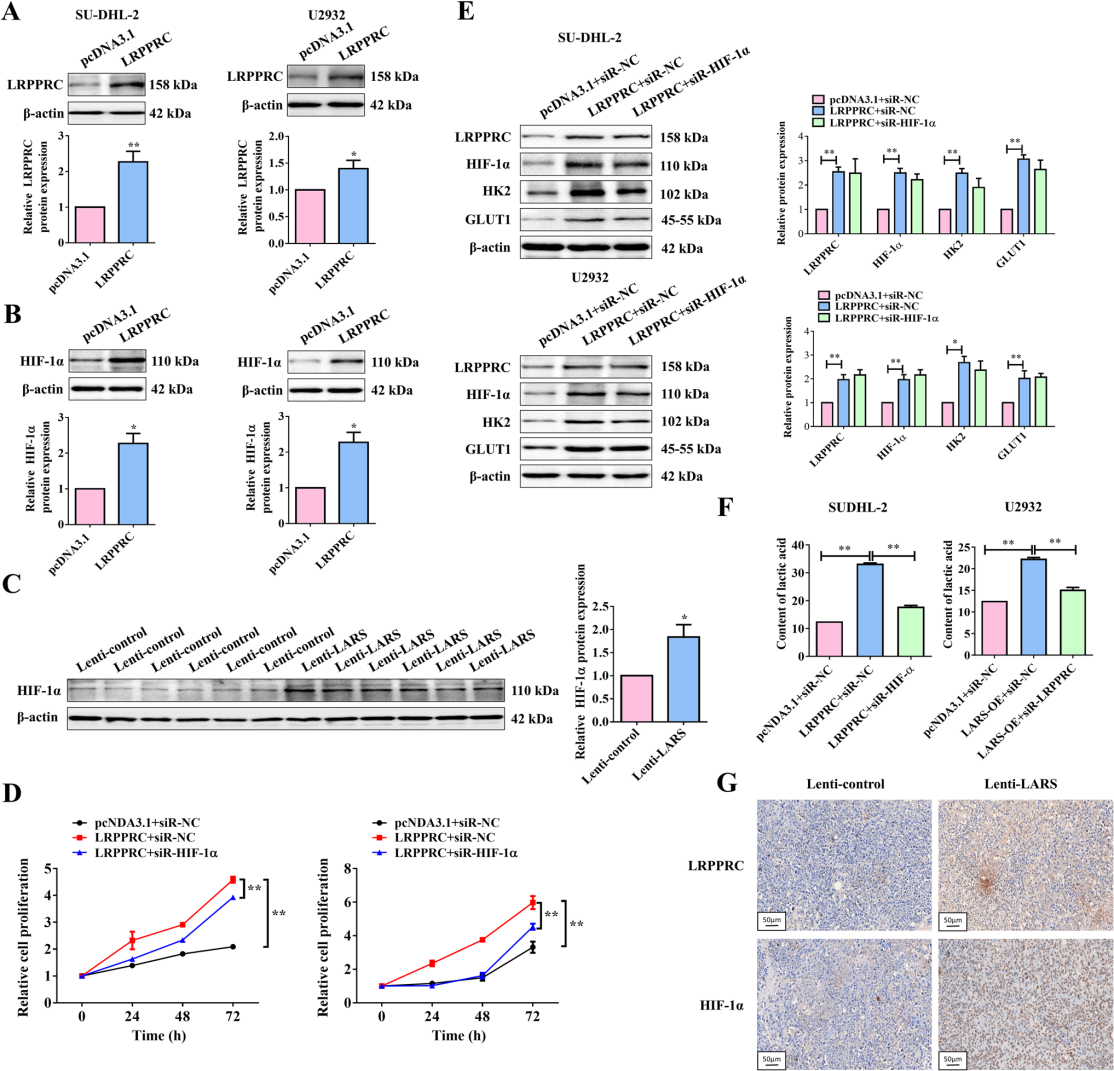
LARS promoted malignant progression by regulating glycolysis via the LRPPRC/HIF-1α axis. (**A**) SU-DHL-2 and U2932 cells were transfected with LRPPRC-overexpressed plasmid or the empty pcDNA3.1 vector, and the overexpressing efficiency was verified by WB assays (*n* = 3). (**B**) The protein expression level of HIF-1α in SU-DHL-2 and U2932 cells overexpressing LRPPRC was detected by WB assays (*n* = 3). (**C**) The protein expression level of HIF-1α in tumor tissues (from Fig. 3B) was examined by WB assays (*n* = 3). (**D**) After transfecting with siR-HIF-1α or siR-NC, the cell proliferation of LRPPRC-overexpressed cells was determined by MTT assays (*n* = 3). (**E**) SU-DHL-2 and U2932 cells were transfected with LRPPRC-overexpressed plasmid or the empty pcDNA3.1 vector, combined with the transfection of siR-HIF-1α or siR-NC. The protein expression levels of LRPPRC, HIF-1α, HK2, and GLUT1 were detected by WB assays. β-Actin served as the loading internal control (*n* = 3). (**F**) The content of LA was examined by ELISA (*n* = 3). (**G**) The expression levels of LRPPRC and HIF-1α from tumor tissues (from Fig. 3B) were detected by IHC assays (*n* = 3). Statistical analysis was performed utilizing a T-test for two-group comparison and one-way ANOVA for multiple-group comparison. *p<0.05, **p<0.01

The above work proved that LARS facilitated glycolysis by regulating LRPPRC expression, but the underlying mechanism needs further research. HIF-1α, a key transcriptional regulator of oxygen homeostasis, exerts an activating influence on the transcription of glucose transporters and glycolytic enzymes, and the subsequent hypoxia-induced glycolysis increase could be utilized by cancer cells for survival and development [20]. Given the close relationship between HIF-1α and glycolysis, and since the KEGG enrichment analysis (Fig. 1D) presented earlier demonstrated that the DEGs were predominantly enriched in the glycolysis/gluconeogenesis and HIF-1 signaling pathway, we recognized the significance of HIF-1α in this study and investigated the association between LRPPRC and HIF-1α. The results indicated that overexpression of LRPPRC led to an increase in HIF-1α expression both in vitro and in vivo (Fig. 5A-C). Consequently, the promoting effect of LARS on the progression of DLBCL, dependent on regulating glycolysis, might be achieved via the LRPPRC/HIF-1α axis. More importantly, overexpression of LRPPRC promoted cell proliferation and inhibited apoptosis in DLBCL cells, whereas these effects could be reversed by the silencing of HIF-1α (Fig. 5D, S1C). The effects of LRPPRC overexpression on the upregulation of HIF-1α, HK2, and GLUT1 expression and the increase in LA content ere attenuated by the silencing of HIF-1α (Fig. 5E&F). Besides, immunohistochemical analysis confirmed that overexpression of LARS upregulated the expression levels of LRPPRC and HIF-1α in tumor tissues (Fig. 5G), consistent with Figs. 4C and 5C.

**Fig. 6:**
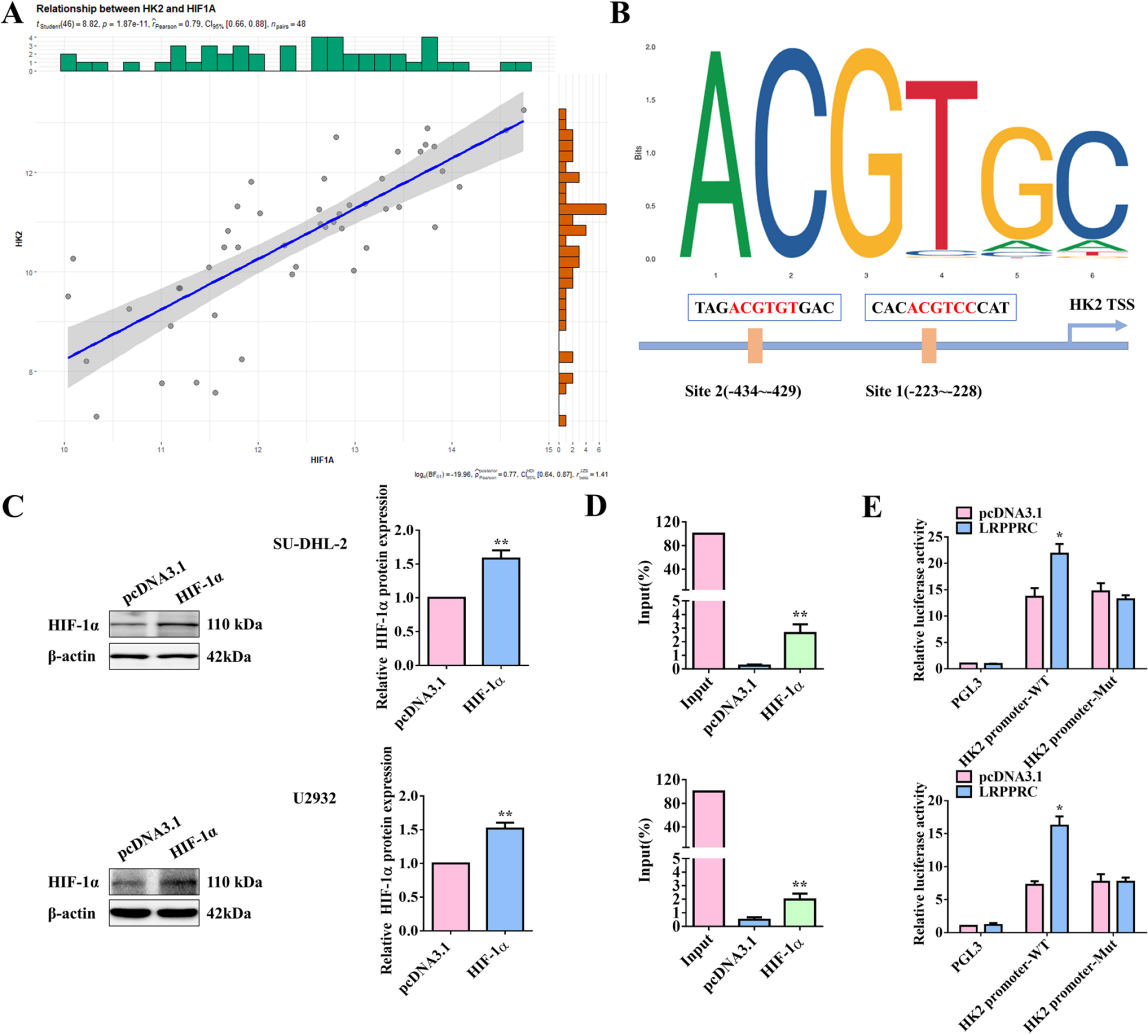
LRPPRC facilitated the transcription of HK2 via HIF-1α. (**A**) A positive correlation between HK2 and HIF-1α. (**B**) HIF-1α had the putative binding sites in the promoter region of HK2. (**C**) SU-DHL-2 and U2932 cells were transfected with HIF-1α-overexpressed plasmid or the blank pcDNA3.1 vector, then the overexpressing efficiency of HIF-1α was verified by WB assays. β-Actin served as the loading internal control (*n* = 3). (**D**) The binding relationship between HIF-1α and the HK2 promoter was verified by CHIP assays (*n* = 3). (**E**) The potential binding site of HIF-1α to the HK2 promoter was mutated in cells overexpressing LRPPRC (*n* = 3). Dual-luciferase reporter assays were used to detect the luciferase intensity of the wild-type or mutant sequence. Statistical analysis was performed utilizing a T-test for two-group comparison and one-way ANOVA for multiple-group comparison. *p<0.05, **p<0.01

A characteristic of tumor cells is their ability to perform glycolysis at a faster rate, with the transcription of the glycolysis rate-limiting enzyme, HK2, serving as a crucial step in this process [21]. Furthermore, HK-2 is one of the downstream transcriptional targets of HIF-1α [22]. For the potential targets, the gene correlation analysis revealed a positive correlation between HK2 and HIF-1α (Fig. 6A). The bioinformatics analysis indicated that HIF-1α had the putative binding sites in the promoter region of HK2 (Fig. 6B). Subsequently, HIF-1α was overexpressed in SU-DHL-2 and U2932 cells (Fig. 6C). The results of chromatin immunoprecipitation (ChIP) demonstrated the binding of HIF-1α to the HK2 promoter (Fig. 6D). Moreover, the dual-luciferase reporter gene assays revealed that the overexpression of LRPPRC promoted the luciferase activity of the HK2 promoter-WT, but had no impact on the luciferase activity of the HK2 promoter-Mut (Fig. 6E). These results are consistent with previous findings, indicating that overexpression of LRPPRC upregulated the expression of HIF-1α and HK2 (Fig. 5E). Therefore, these findings suggested that LRPPRC upregulated the expression level of HIF-1α, which bound to HK2 and promoted its transcription.

In summary, LARS promoted glycolysis through the LRPPRC/HIF-1α/HK2 axis, ultimately driving the malignant progression of DLBCL.

## Discussion

According to the Warburg effect, the rapid growth rate of tumor tissues depends on efficient energy metabolism. Tumor tissues carry out glucose metabolism much more efficiently than normal tissues even in aerobic conditions [23]. Glycolysis, the critical process of glucose metabolism, has been proven to serve as the main source of energy for the majority of cancer cells [24]. More importantly, glycolysis plays a pro-cancer role in multiple cancers, for instance, enhanced glycolysis provides energy for the growth and metastasis of pancreatic cancer [25]. Besides, glycolysis contributes to the cell survival and proliferation of breast cancer cells [26]. LARS studied in this work, is a key leucine sensor that plays a vital role in the connection of leucine and the homologous tRNA and conduces to the signal transduction of leucine [27]. It has been reported that leucine inhibits glycolysis, while LARS uses dissociative leucine to promote glycolysis [10]. Intriguingly, the impact of LARS on diverse cancer progression is complicated. A previous study indicated that LARS is conducive to the germination and migration of lung cancer [28], while another one found that it inhibited the tumourigenesis and proliferation of breast cancer [29]. Given these data, LARS may serve as an effective therapeutic target for cancer treatment, which may be related to glycolysis, yet its underlying functional mechanism in DLBCL needs to be investigated.

Indeed, in this study, we demonstrated that LARS promoted the malignant progression and glycolysis of DLBCL both in vitro and in vivo. LARS facilitated cell proliferation but inhibited apoptosis in SU-DHL-2 and U2932 cells. Meanwhile, LARS promoted tumor growth and raised the expression of LA (the product of glycolysis) and that of HK2, GLUT1, and HIF-1α, the key enzymes in glycolysis, which were reversed upon 2-DG treatment. The LARS knockdown experiment further supported our conclusion. Furthermore, we also discovered that LARS upregulated the LRPPRC expression at the protein level while not affecting the mRNA level. It has been reported that LARS also serves as the translational regulator that affects the translation of some leucine-rich proteins, tumor-associated proteins, and the downstream proteins of LARS in a codon-dependent manner [29]. Other reports also demonstrated the crucial role of LARS in protein synthesis [30], which may explain why LARS affected the LRPPRC expression only at the protein translation level.

LARRPC is a multifunctional protein that is closely related to the malignant progression of multiple cancers [19]. It has been proven that LRPPRC promoted invasion, but inhibited apoptosis in prostate cancer, lymphoma, lung adenocarcinoma, and so on [31, 32]. Likewise, LRPPRC promoted cell proliferation, metastasis, and invasion of pancreatic cancer cells [33]. Interestingly, LRPPRC has also been confirmed to be conducive to the drug resistance of tumors [34, 35]. In our work, we mainly focused on the influence of LRPPRC on the malignant progression and glycolysis of DLBCL cell lines. We found that the promoting effect of LARS on the DLBCL malignant phenotypes, glycolysis-related proteins, and the glycolytic intermediates LA could be alleviated by the silenced LRPPRC. Importantly, rescue experiments where LRPPRC overexpression reversed the malignant phenotypes of LARS knockdown further corroborate our hypothesis of a LARS–LRPPRC functional axis in DLBCL pathogenesis. Immunohistochemical analysis further confirmed that overexpression of LARS upregulated the expression levels of LRPPRC and HIF-1α in vivo, illustrating the indispensable role of LARS in regulating glycolysis to boost the progression of DLBCL.

Cancer progression is also mediated by some transcription factors, such as HIF-1α. According to reports, the rapid proliferation of tumors leads to hypoxia in the tumor microenvironment (TME), which activates the activity of HIF-1α, the crucial transcriptional factor that helps upregulate cancer-associated proteins and promote tumor progression [36]. More importantly, the metabolism reprogramming of cancer cells drives a shift in mitochondrial respiration toward glycolysis to obtain sufficient energy supply under hypoxia conditions, HIF-1α helps upregulate the expression of glycolysis key enzymes and glucose transporters in this process [20]. Hence, we speculated that HIF-1α might promote cancer progression by regulating glycolysis.

Back to our study, we found that LARS and LRPPRC played an upregulating role in HIF-1α expression. More importantly, the promoting effect of the overexpressed LARS on the DLBCL progression and glycolysis could be reverted by the silenced HIF-1α. Indeed, the subsequent luciferase reporter and CHIP assay confirmed the binding site of HIF-1α of the HK2 promoter, which further demonstrated the underlying mechanism by which LARS positively regulated DLBCL progression. It has been reported that LRPPRC promotes retinoblastoma progression and glycolysis by targeting HIF-1α [37], which is corroborates with our results. Specifically, LARS positively regulated glycolysis, targeting the LRPPRC/HIF-1α axis, which subsequently boosted malignant progression in DLBCL.

Building on our bioinformatics-driven discovery, experimental validation confirmed LARS as a functional metabolic regulator in DLBCL via the LRPPRC/HIF-1α/HK2 axis. However, several limitations temper the translational potential of these findings. Although single-cell RNA-seq analysis revealed a consistent trend of elevated LARS expression in DLBCL samples compared to adjacent normal controls, we lacked paired clinical samples for protein-level validation. Additionally, the prognostic significance of LARS expression remains inconclusive due to inherent constraints of bulk transcriptomic analyses and cohort heterogeneity in available datasets. Future studies should prioritize developing robust immunohistochemical assays for LARS detection in clinical specimens, combined with spatial multi-omics approaches to delineate its cellular distribution within tumor microenvironments. Complementary investigations using patient-derived models will be critical for assessing the therapeutic vulnerability of this metabolic pathway, particularly in the context of existing treatment regimens. Together, these efforts will be essential for translating our mechanistic insights into clinically actionable DLBCL management strategies.

## Conclusion

Our study confirmed that LARS is deeply associated with the progression of DLBCL. Mechanistically, LARS facilitated glycolysis via the LRPPRC/HIF-1α/HK2 axis, thus propelling the malignant progression of DLBCL. The data in this work provided a theoretical basis for LARS as a potential therapeutic target for the treatment of DLBCL.

## Supplementary Information

Below is the link to the electronic supplementary material.

Supplementary file1 (DOCX 6722 kb)

## Data Availability

The data generated or analyzed during this study are shown in this published article.
