# Supplementary material for: Leucyl-tRNA synthetase promotes malignant progression in diffuse large B-cell lymphoma by regulating glycolysis via the LRPPRC/HIF-1α/HK2 axis
Source: Hum Cell. 2025 Aug 7;38(5):139. doi: 10.1007/s13577-025-01267-y (PMC12331823; doi:10.1007/s13577-025-01267-y)
Supplement: Supplementary file 1 — Supplementary file1 (DOCX 6722 kb) [file 13577_2025_1267_MOESM1_ESM.docx]

**
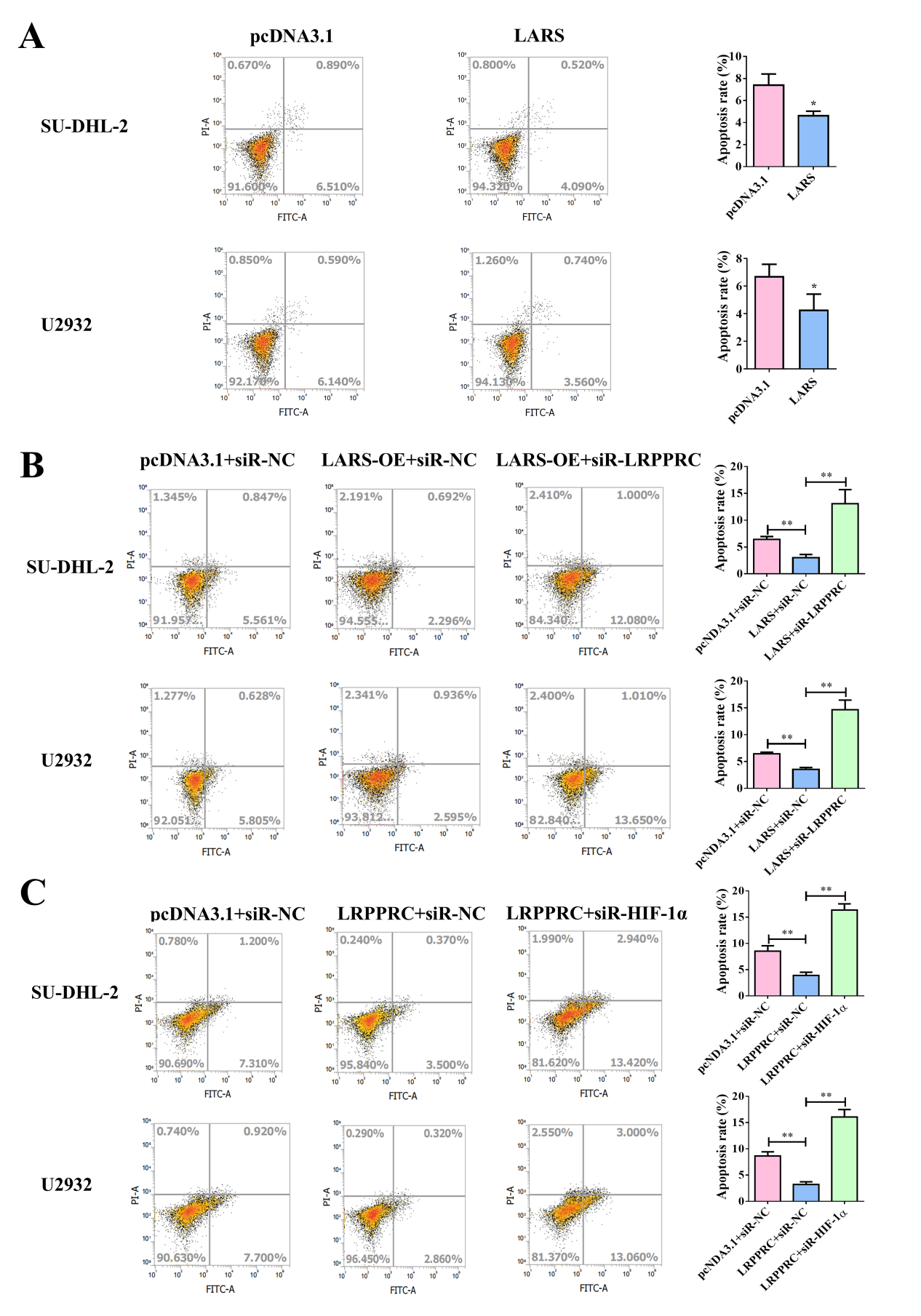
Fig.S1** Apoptosis in different conditions.

(A) The apoptosis of the two DLBCL cell lines overexpressing LARS was detected by flow cytometry (n=3). (B) The apoptosis of the LARS-overexpressed DLBCL cell lines (SU-DHL-2 and U2932 cells) transfected with siR-LRPPRC or siR-NC was examined by flow cytometry (n=3). (C) The apoptosis of the LRPPRC-overexpressed DLBCL cell lines (SU-DHL-2 and U2932 cells) transfected with siR-HIF-1α or siR-NC was examined by flow cytometry (n=3). Statistical analysis was performed utilizing a T-test for two-group comparison and one-way ANOVA for multiple-group comparison. **p*<0.05, ***p*<0.01
